# Early life movements and mortality of Egyptian vultures: Implications for transcontinental conservation

**DOI:** 10.1002/ece3.70291

**Published:** 2024-09-15

**Authors:** Juan Oltra, Javier García, Isidoro Carbonell, José Jambas, Ernesto Álvarez, Juan J. Iglesias‐Lebrija, Alberto Gil‐Carrera, Juan M. Pérez‐García, Óscar Frías, José L. González del Barrio, Guillermo Blanco, Martina Carrete

**Affiliations:** ^1^ Department of Physical, Chemical and Natural Systems Universidad Pablo de Olavide Sevilla Spain; ^2^ Grupo Ibérico de Anillamiento León Spain; ^3^ SALORO S.L.U Salamanca Spain; ^4^ Oriolus Ambiente e Eco Turismo LDA Oporto Portugal; ^5^ Grupo de Rehabilitación de la Fauna Autóctona y su Hábitat (GREFA) Majadahonda Spain; ^6^ Estació Biolóxica do Xurés Lobios Galicia Spain; ^7^ Department of Applied Biology Miguel Hernández University Elche Spain; ^8^ Department of Evolutionary Ecology Museo Nacional de Ciencias Naturales (CSIC) Madrid Spain

**Keywords:** dependence period, Egyptian vulture, Iberian Peninsula, migration, mortality, Sahel

## Abstract

Understanding the movements and mortality of individuals across different life stages is crucial for the effective conservation of wild populations. We used data from 32 Egyptian vultures (*Neophron percnopterus*) tagged with GPS transmitters as nestlings in three Iberian breeding areas to study their dependence period, migration routes, movements in Africa, and mortality at each stage. Our results show no significant differences in the timing of nest departure or the duration of the dependence period between individuals of different sexes or breeding nuclei. Most juveniles migrated to sub‐Saharan Africa in their first year, but some (3 of 32, 9.4%) remained in the Iberian Peninsula. Individuals that migrated to Africa did so annually, while those remaining in Iberia never migrated to the Sahel, indicating distinct migratory and non‐migratory strategies. Non‐migratory individuals consistently moved northward during the breeding season to their natal territories. Siblings did not coordinate their migration strategy or timing. All juveniles showed extensive overlap in the vast areas used in Africa, where females arrived before males, and in the Iberian Peninsula. Our study also revealed that no juveniles died immediately after fledging, but that none of the tagged individuals lived more than 7 years or were recruited as breeders. Although most casualties occurred during the longer stay in the Sahel, the mortality rate was highest during the few days of the first migration. Our results show that despite small variations in movement patterns between breeding nuclei and sexes, Egyptian vultures face similar challenges during the years before recruitment as breeders, mostly determined by their migratory strategy. These findings are relevant for designing conservation strategies, both in breeding areas and, more importantly, in wintering areas and along migration pathways. Such strategies will significantly impact the entire Iberian population of this endangered species.

## Graphical abstract

We used data from 32 Egyptian vultures (*Neophron percnopterus*) tagged with GPS transmitters as nestlings in three Iberian breeding areas to study their dependence period, migration routes, movements in Africa, and mortality at each stage. Our results show no significant differences in the timing of nest departure or the duration of the dependence period between individuals of different sexes or nuclei. Most juveniles migrated to sub‐Saharan Africa in their first year, but some (3 of 32, 9.4%) remained in the Iberian Peninsula. Individuals that migrated to Africa did so annually, while those remaining in Iberia never migrated to the Sahel, indicating distinct migratory and non‐migratory strategies.

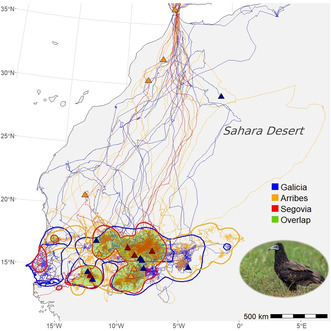

## 1 INTRODUCTION

Birds exhibit enormous interspecific variability in their life histories, which affects the process by which offspring acquire independence (Mainwaring, 2016). Parental duties during the period of offspring dependence are variably prolonged depending on the position of each species on the altricial–precocial continuum, and due to intraspecific factors (Ar & Yom‐Tov, 1978; Ducatez & Field, 2021). Juveniles then go through a period of transition to independence, one of the most important stages in an individual's life. This is due to the fact that their inexperience in foraging and avoiding predators, in addition to anthropogenic risk factors, drastically increases their mortality risk (Naef‐Daenzer & Grüebler, 2016). In raptors, where parental care is generally prolonged, the duration of the dependence period has been evaluated by considering parent–offspring trade‐offs modulated by factors such as climate, predation risk, competence, or food availability (Ceballos & Donázar, 1990; Newton, 2010; Tapia & Zuberogoitia, 2018). Previous studies have shown that food availability and predictability in breeding grounds influence the behavior and movement range of juveniles during their dependence period (Newton, 2023). Thus, spatiotemporal variability in these factors may contribute to differences in the duration of the dependence period between breeding nuclei or populations, affecting the strategies of individuals (movements), their mortality or the severity with which non‐lethal effects condition them in future stages (carry‐over effects; Newton, 2024; Norris, 2005).

After becoming independent, individuals move away from their natal areas for months or even years before establishing in a breeding site (Newton, 2010). During this period, they must acquire the skills necessary for survival, including the use of cues to identify temporally and spatially dispersed resources and suitable breeding areas for settlement (Greenwood & Harvey 1982; Newton, 2010; Ronce, 2007). For migratory species, individuals often face their first, long, and sometimes virtually non‐stop migration just after leaving their birthplace (Neubauer et al., 2012; Rotics et al., 2016). These individuals are exposed to greater potential risks than older individuals that have already undergone this experience, resulting in significantly lower survival rates (Newton, 2024). Factors such as the timing of hatching or nest departure, the duration of the dependence period or the quality of the natal territory may influence the state in which juveniles face their first migration and lead to marked differences in their survival (Newton, 2010; Tapia & Zuberogoitia, 2018). This information on possible population and sex variations in the dependence period, migration, use of space, and survival of individuals in their pre‐recruitment stages is particularly scarce in long‐lived species such as vultures, despite its relevance for the management and conservation of their populations.

The Egyptian vulture (*Neophron percnopterus*) is one of the smallest Old World vultures inhabiting deserts, steppes, and mountains in Africa, Europe, and Asia. In the western part of their distribution, the species behaves as a trans‐Saharan migrant in continental Europe and as a resident in the Spanish archipelagos (Oppel, Arkumarev, et al., 2021; Oppel, Buechley, et al., 2021), although it has recently been documented as a year‐round resident in the Iberian Peninsula (Morant et al., 2020, 2022). This species is listed as “Vulnerable” in Europe due to population declines in recent decades (BirdLife International, 2021) related to high mortality rates of adults and juveniles in breeding areas and during migration and stay in the Sahel (Africa), respectively (BirdLife International, 2021). Previous studies have described the migration of subadult and adult Egyptian vultures along the western European‐African flyway, showing a large overlap between individuals in the Sahel (García‐Ripollés et al., 2010; López‐López et al., 2014). However, the independence period and migration of juveniles born in different breeding nuclei have not been investigated, nor has it been studied whether they converge or segregate during their stay in Africa. It also remains unexplored whether factors such as events during the dependence period, individual characteristics (e.g., sex), or kinship relationships influence the movements or migratory patterns of these inexperienced individuals.

Here, we assess the effects of individual characteristics, in particular sex and natal areas, on the duration of the dependence period, migratory movements, and stay in Africa and the Iberian Peninsula of juvenile and subadult Egyptian vultures. We tested the hypothesis that movements and mortality of individuals in their early life stages are influenced by local conditions in breeding nuclei and by intrinsic individual traits, such as age and sex. The effects of local conditions can influence movements and survival directly (e.g., through climatology, food availability, or intra‐ and interspecific competence) or indirectly through their effects on condition (“silver spoon effect”; Van De Pol et al., 2006). These effects are expected to homogenize with age, as individuals use areas other than those of birth and gain experience. Furthermore, previous studies show that movements and areas prospected by males and females differ, both in this (Serrano et al., 2021) and other vulture species (García‐Macía et al., 2024; Margalida et al., 2016; Morant et al., 2023). Therefore, we also expect differences in movements related to sex and age of individuals, which could lead to differences in mortality between males and females and between younger and older individuals. All this knowledge will enhance our understanding of the constraints affecting individuals during their early life stages, providing essential information for the conservation of this endangered species.

## 2 MATERIALS AND METHODS

### 2.1 Study areas

**FIGURE 1:**
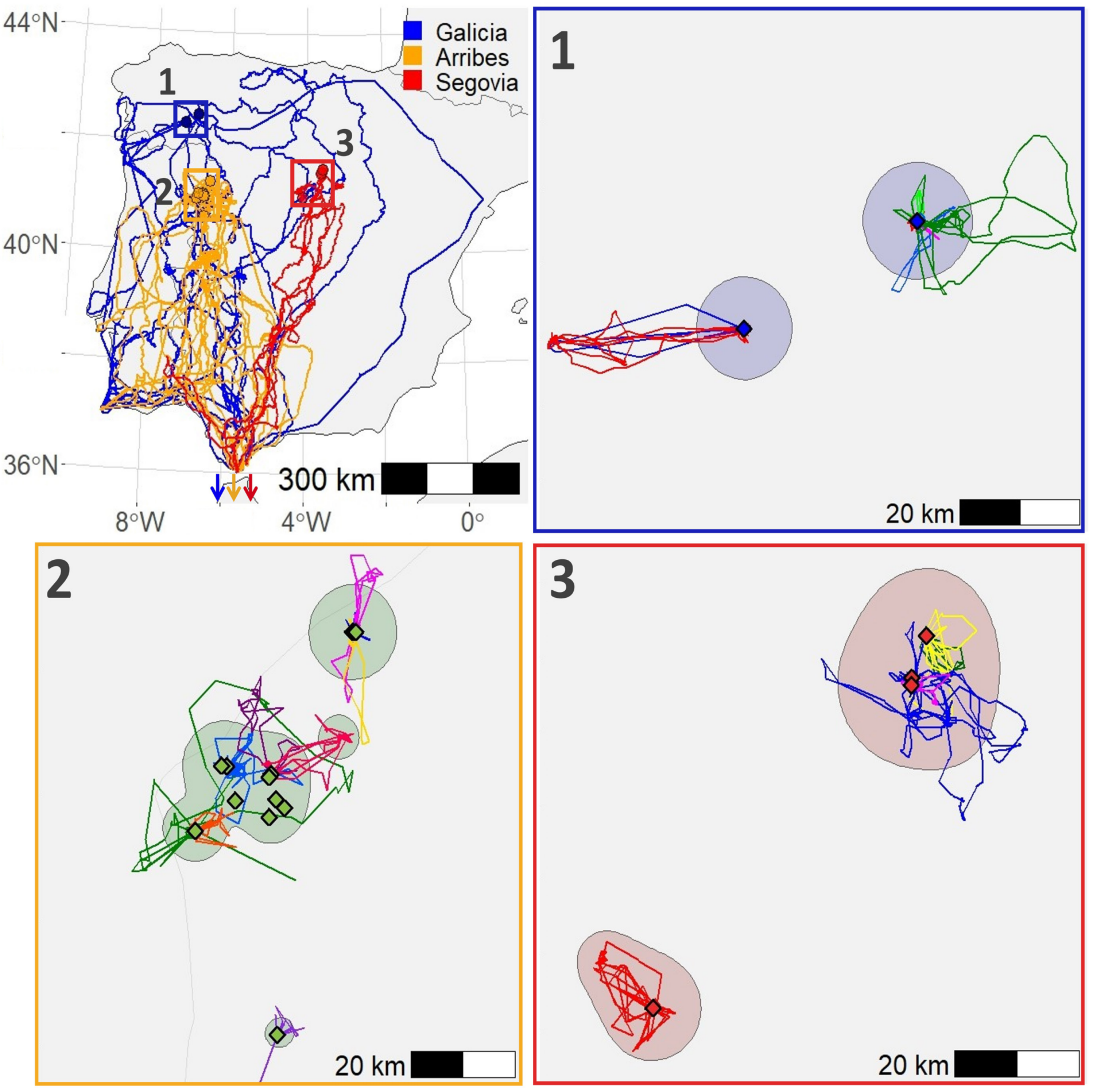
Movements across the Iberian Peninsula of GPS‐tagged juvenile Egyptian vultures *Neophron percnopterus* born in three Spanish breeding nuclei, namely, Arribes del Duero (orange symbols), Galicia (blue symbols), and Segovia (red symbols). Panels 1–3. Movements of individuals during the dependence period. The shaded area represents the KDE 95% area. Dots represent the different nests where the individuals were tagged and the different colors of the lines on these panels represent the movements of the different individuals.

Egyptian vultures were tagged in three breeding nuclei located in central and northern Spain (Figure 1). The Galician nucleus represents the northwestern limit of the Iberian range of the species, with only two active breeding territories according to the most recent national census of 2018 (Del Moral & Molina, 2020). These breeding pairs live in areas with a Mediterranean climate, with the presence of large forest ungulates and extensive livestock, without supplementary feeding points for necrophagous birds. The Arribes del Duero nucleus (hereafter Arribes, provinces of Salamanca and Zamora) has a high density of breeding pairs (114 breeding pairs in 2018) nesting in canyons along the Duero River, on the border between Spain and Portugal. The trend in the number of breeding pairs in this nucleus points to a slight general decrease in the recent decades (Junta de Castilla y León, 2023). The area is dominated by dehesas with high livestock densities. The Segovian nucleus is located on the banks of the Riaza and Duratón rivers, with isolated pairs on the small cliffs that surround them (about 30 breeding pairs in 2018). The habitat consists of moorland areas and dry crops. The main diet of Egyptian vultures in this area is based on carcasses of intensive livestock (mainly pigs provided at supplementary feeding points) and wild animals (Blanco & Hornero‐Méndez, 2023). This nucleus has shown a decreasing trend in recent decades due to several factors, including poisoning, inbreeding, contamination, and disease (Blanco et al., 2017; Blanco & Morinha, 2021; Pitrach et al., 2017).

### 2.2 Fieldwork

Between 2015 and 2023, a total of 32 nestling Egyptian vultures were ringed and fitted with GPS transmitters, 17 in Arribes, 10 in Galicia, and 5 in Segovia. The GPS‐tagged Egyptian vultures were sexed to 16 males and 16 females by molecular methods following Fridolfsson and Ellegren (1999) using a drop of blood from the brachial vein. The nests were accessed when nestlings had reached adult size but were not yet able to fly. In both the Arribes and Segovia areas, four transmitters were fitted on siblings (one pair of siblings in each nucleus). All individuals from Arribes and Segovia and two from Galicia were fitted with solar‐powered GPS‐GSM (30 g; Ornitela, Vilnius, Lithuania; www.ornitela.com). Five from Galicia were tagged with E‐obs devices (48 g; E‐Obs GMBH, Gruenwald, Germany). Two birds were tagged with PPT‐100 GPS/Argos Solar MTI (45 g; Microwave Telemetry Inc., Columbia, Maryland, USA) and one bird was tagged with Ecotone (33 g; Ecotone Telemetry, Gdynia, Poland) (Table S1). Transmitters were attached to the backs of the nestlings using harnesses (6 mm Teflon ribbon) (Thaxter et al., 2014) and programmed to record fixes (i.e., GPS positions) at flexible schedules between 5 and 30 min, depending on battery charge (mean interval between positions: 30.5 min, SD: 132.7 min). The weight of the transmitters was below 3% of the body weight of the tagged individuals (mean body mass = 1861.5 g, SD: 198.1 g), thus adhering to recommended limits to avoid adverse effects on the birds (i.e., 3% body mass threshold, see, e.g., Sergio et al., 2015). GPS data were incorporated into the Movebank online data repository (www.movebank.org; Kays et al., 2022).

### 2.3 Bird movements

Our database included all GPS locations of individuals from the date of tagging at the nest to the last location emitted by the transmitters, either because its lifetime expired or because the individual had died. In cases where individuals were still alive, the last location corresponds to May 1, 2024. These data allowed us to focus on the study of movements during the first years of life of Egyptian vultures (their dependence period, migration, and first return to the Iberian Peninsula, or for the first 2 years in the case of non‐migratory individuals to make information comparable due to the different years of tagging). Moreover, they also provided us with more general information on the activity and survival of Egyptian vultures before their recruitment into the breeding population. Data with high GPS error were filtered by excluding fixes with VDOP (Vertical Dilution of Precision) and HDOP (Horizontal Dilution of Precision) > 5 and resampled to standardize the time between consecutive fixes to 30 min.

We determined the timing, duration, and movements made by each individual during the dependence period (i.e., from leaving the nest to leaving the natal territory), as well as its movements during the migration period (i.e., from leaving the natal territory to arriving at the Sahel grounds, differentiating the stay in the Iberian Peninsula and in Africa) and the stay in Africa (i.e., from arriving at the Sahel to the beginning of the return to Europe). The date of leaving the nest was estimated as the first time each individual moved a distance of 200 m or more. The date of departure from the natal territory was estimated as the time when individuals stopped returning to the nest, which consistently coincided with long and clearly directed journeys, generally heading southwards. The trajectories of each individual were used to calculate the longitudinal distance travelled (migration) by summing the linear segments between consecutive GPS fixes and the area covered by the birds during each stage. Following Oppel, Arkumarev, et al. (2021), we estimated the area used by each individual in the Sahel using the kernel density estimator at the 95% isopleth contour (95% KDE) of all locations of individuals until they left it to migrate northward again (clearly directed latitudinal displacements). To assess the possible segregation of individuals born in different breeding nuclei in the Sahel, we calculated the overlap (in percentage) and the distance between the centroids of the 95% KDE for each pair of individuals. For non‐migratory individuals, we calculated the monthly 95% KDE for locations collected since leaving the natal territory. Trajectories and KDE were obtained using the amt package (Signer et al., 2019) in statistical software R 4.3.2 (R Core Team, 2023).

We estimated mortality at each stage considering that a vulture died when the GPS signal was lost abruptly, despite good transmission and battery performance, or when the transmitter emitted continuously from the same position and accelerometers stabilized, indicating that the animal's body was not moving (Sergio et al., 2018). As most individuals disappeared in Africa, we were unable to conduct any additional field research to assess if they were actually dead and the causes. For individuals that carried transmitters for more than 5 years and that, after that period, started to emit intermittently, we considered it was alive at the last location received before such irregular reception.

### 2.4 Statistical analysis

We used linear mixed models (LMM, glmmTMB package; Brooks et al., 2017) to assess whether the timing (Julian date, Julian day number 1 assigned to 1st of January) and duration (number of days) of the dependence period, migration and stay in the Sahel, as well as the distance travelled and the total area covered during each stage, differed between individuals born in the three breeding nuclei and between males and females (fixed factors). Similarly, we compared the overlap of Sahelian areas and the distance between their centroids between males and females born in the same and different breeding nuclei (fixed factors), and the surface of the areas where birds stayed in Africa with those of the non‐migrants that stayed in the Iberian Peninsula in the same period. In all cases, the year of tagging of each individual was included as a random factor to correct for interannual differences and reduce pseudo‐replication. Whenever necessary, the dependent variables were logarithmically transformed to normalize the data (see variable details in Table S2). When significant effects were found (*p* < .05), post hoc tests were performed to determine which groups differed using the *lsmeans* package in R (Lenth, 2016), adjusting for multiple comparisons with Tukey's method. The goodness of fit of the final models was assessed using the Dharma R package (Hartig, 2022) in R.

The best way to estimate the survival of individuals is to use capture–recapture models, which allow incorporating the uncertainty associated with possible transmitter failures (Arrondo et al., 2020). However, this approach has not been possible due to the limited sample of animals tagged. Thus, we estimated survival curves using the Kaplan–Meier method to compare the mean survival of birds between sexes and breeding nuclei (log‐rank tests). One of the fundamental assumptions of this method is that the status of individuals (dead or alive) is accurately known. Although three GPS devices stopped transmitting at some point during the birds' stay in the Sahel, the signals were recovered when these individuals migrated northward, either upon reaching North Africa or upon reaching the Iberian Peninsula. Therefore, we assume that the complete loss of signal reception from a device was not due to electronic failures and was assimilated to mortality (Sergio et al., 2019). The other transmitter that gave problems did so intermittently at the end of its lifetime. Due to these irregularities, we excluded all data collected thereafter. As we aim to contribute to understanding the mortality of these long‐lived species during the early stages of their lives, we have considered the complete information of the individuals, from their tagging to the last date of signal reception or to the present, covering a maximum total period of more than 6 years (although most of the data are temporally shorter due to bird loss or the half‐lives of each transmitter model). All statistical analyses were performed with R 4.3.2 (R Core Team, 2023).

## 3 RESULTS

### 3.1 Dependence period

Individuals from Arribes and Galicia left their nests at the end of July, while birds from Segovia did so at the beginning of August, without significant differences between nuclei (*χ* ^2^ = 1.78, df = 2, *p* = .411). Likewise, all individuals left their natal territories on similar dates (*χ* ^2^ = 2.47, df = 2, *p* = .291), and therefore, there was no significant differences in the duration of the dependency period between breeding nuclei (21.30 ± 12.74 days in Galicia, 19.88 ± 15.32 days in Arribes, and 16.40 ± 5.55 days in Segovia; *χ* ^2^ = 0.48, df = 2, *p* = .786).

During the dependence period, individuals travelled an average of 10.3 km per day (range: 0.06–32.95 km). The maximum distance at which the birds moved (mean maximum distance ± SD: Galicia: 12.38 ± 13.18 km, Arribes: 8.42 ± 9.39 km, Segovia: 12.46 ± 6.53 km; *χ* ^2^ = 0.74, df = 2, *p* = .691), and the prospected areas (mean area ± SD: Galicia: 98.5 ± 246.0 km^2^, Arribes: 22.4 ± 61.5 km^2^, Segovia: 75.3 ± 77.2 km^2^; *χ* ^2^ = 4.98, df = 2, *p* = .083) were similar among the three nuclei.

No significant differences were found between males and females on the date of departure from the nest (*χ* ^2^ = 0.55, df = 1, *p* = .458) or natal territory (*χ* ^2^ = 0.07, df = 1, *p* = .785), nor in the maximum distance (*χ* ^2^ = 0.03, df = 1, *p* = .855) or the area travelled (*χ* ^2^ = 0.29, df = 1, *p* = .592).

### 3.2 Migration

Egyptian vultures left their natal territories and moved across the Iberian Peninsula to migrate to their African quarters throughout the Strait of Gibraltar, with the exception of three individuals from Arribes (one male and two females) that remained in the dehesas of western Spain. These individuals did not migrate to Africa in the following years either, but remained on the Iberian Peninsula as year‐round residents.

All migrants (29 individuals) stayed in the Iberian Peninsula for an average of 15.0 ± 11.7 days since leaving their natal territories (range: 2–59 days, comparison between breeding nuclei: *χ* ^2^ = 2.94, df = 2, *p* = .230; comparison between sexes: *χ* ^2^ = 2.99, df = 1, *p* = .084), travelling a mean distance of 2083 ± 1264.3 km (*χ* ^2^ = 6.82, df = 2, *p* = .033; a posteriori tests: Arribes–Galicia: *p* = .053, Arribes–Segovia: *p* = .995, Galicia–Segovia: *p* = .190). Individuals from Galicia showed the longest (Galicia: 2824 ± 1829 km, Arribes: 1647 ± 580 km, Segovia: 1734 ± 335 km) and least direct routes (Figure 1).

**FIGURE 2:**
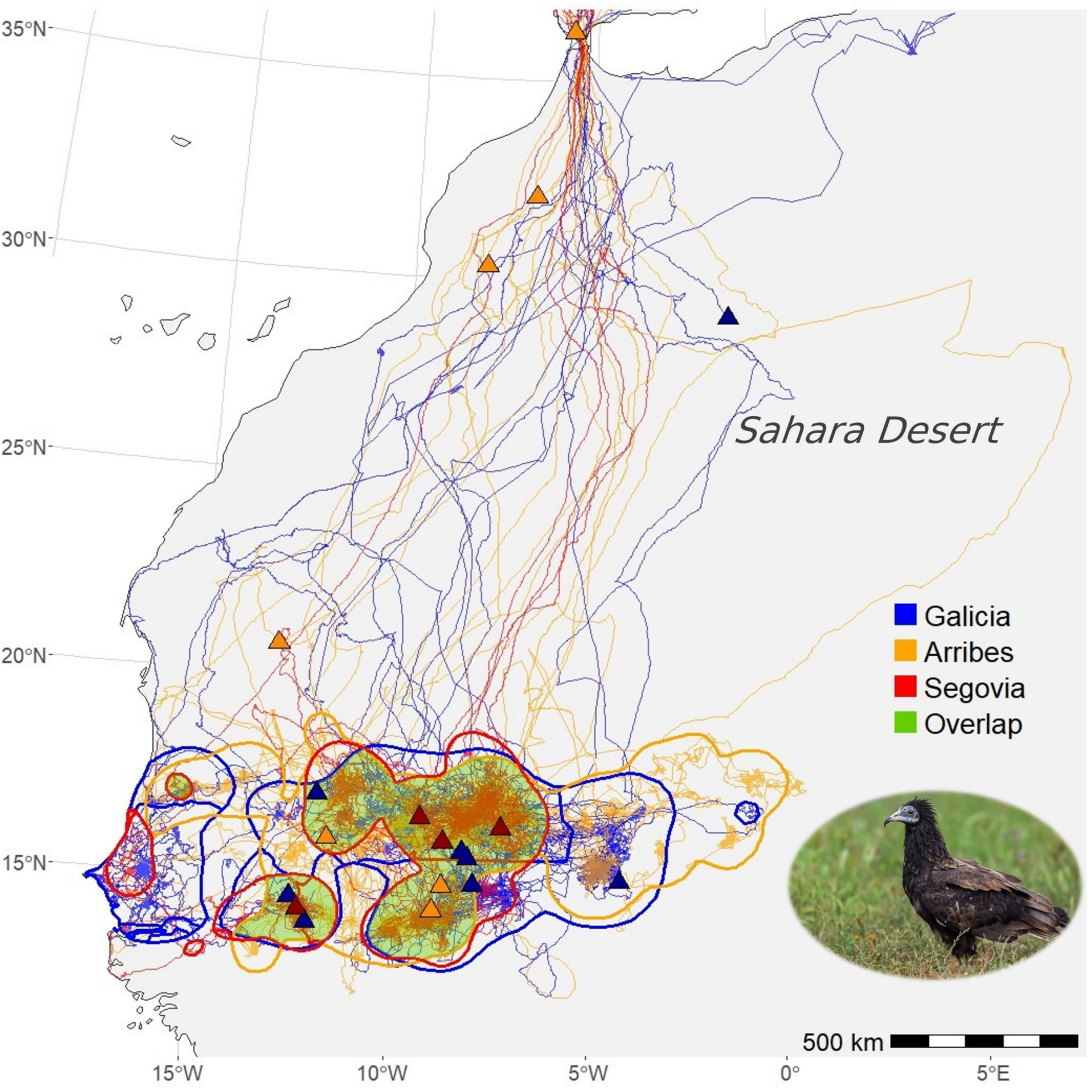
Movements of GPS‐tagged juvenile Egyptian vultures *Neophron percnopterus* born in three Spanish breeding nuclei, namely Arribes del Duero (orange symbols), Galicia (blue symbols) and Segovia (red symbols). Triangles show the location where individuals presumably died. Polygons delineate the Sahel areas utilized by individuals born in the different Spanish breeding nuclei. The green shading represents the 95% KDE obtained using the positions of all individuals, regardless of their natal nuclei.

With the exception of one juvenile born in Arribes in 2023, which died near the Strait of Gibraltar on the Spanish side, the other 28 migrants crossed to Africa between late August and mid‐October. Although the differences were marginally significant, females spent less time in Iberia (*χ* ^2^ = 2.99, df = 1, *p* = .084) and crossed the Strait of Gibraltar to Africa earlier than males (*χ* ^2^ = 3.30, df = 1, *p* = .070). Juveniles traversed the Sahara Desert in 11 ± 5.1 days (range: 6–28 days; comparison between breeding nuclei: *χ* ^2^ = 4.67, df = 2, *p* = .097; comparison between sexes: *χ* ^2^ = 0.91, df = 1, *p* = .341; Figure 2), reaching the Sahel between mid‐September and early November, with no differences between nuclei (*χ* ^2^ = 2.08, df = 2, *p* = .354). Females arrived at the Sahel earlier than males (estimate for females: 265.8 ± 4.75, *χ* ^2^ = 4.61, df = 1, *p* = .034).

The two siblings from Arribes (one male and one female) followed completely different strategies: While the female remained in the Iberian Peninsula, the male migrated to sub‐Saharan areas. The Segovian siblings (one male and one female) migrated to Africa along the same route, but 3 days apart. Individuals from Galicia are all from the only two territories in the area, but the adults were not tagged so we do not know for sure if they were siblings from different breeding seasons.

### 3.3 Stay in the Sahel and permanency in the Iberian Peninsula

Juveniles migrating to Africa moved over huge areas (mean: 160,056 km^2^; range: 21,427–562,507 km^2^) between the coast of Senegal and eastern Mali (between 13.3° and 22.5° latitude, Figure 2). This vast Sahelian region was shared by individuals of the three breeding nuclei (32% and 30% overlap for individuals from the same and different nuclei, respectively; *χ* ^2^ = 0.97, df = 1, *p* = .325; 410 and 419 km between the centroids of the areas used in the Sahel by individuals from the same and different nuclei, respectively; *χ* ^2^ = 0.48, df = 1, *p* = .488).

**FIGURE 3:**
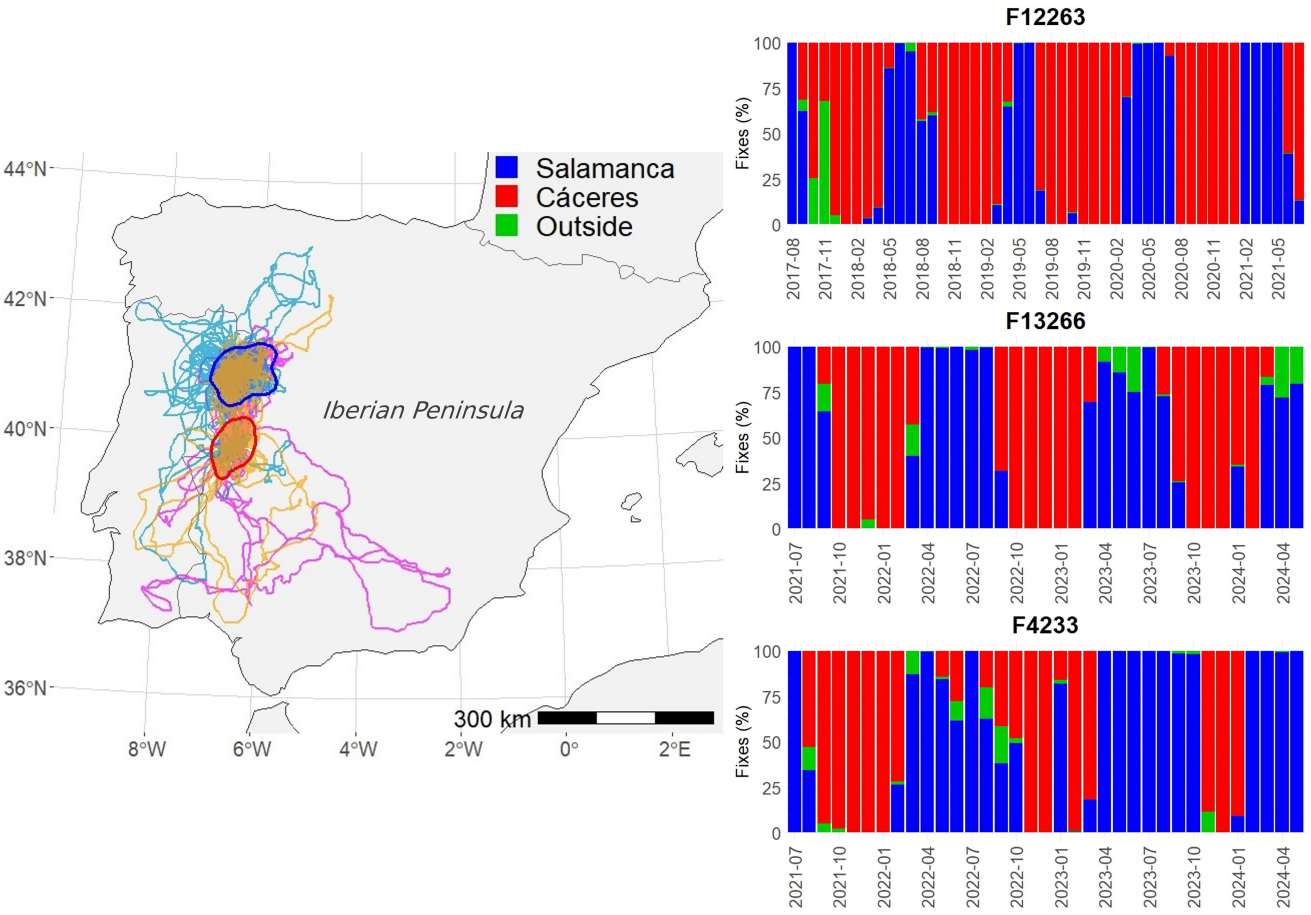
Movements of the three Egyptian vultures *Neophron percnopterus* born in Arribes del Duero that remained resident in the Iberian Peninsula throughout their lives (each color lines represent an individual). The polygons delineate the two core areas used by these individuals (blue, Salamanca; red: Cáceres), obtained as the 95% KDE of all their locations since they left their natal territories. Histograms on the right represent the monthly percentage of GPS fixes of each individual within each core area (blue, Salamanca; red: Cáceres). Green bars represent pooled data of movements of the three individuals outside the two core areas.

The three individuals that did not migrate to Africa moved around two cores located in the regions of Cáceres and Salamanca, in western Spain (Figure 3), covering much smaller areas (mean: 14,870 km^2^; range: 10,224–19,026 km^2^) than those reaching the Sahel (see above, *χ* ^2^ = 14.92, df = 1, *p* < .001). The three non‐migratory individuals followed a similar movement pattern between the two core areas, staying in southern area (Cáceres) from autumn to spring (approximately September–March) and moving northward (Salamanca) during the breeding season (approximately April–August) (Figure 3).

### 3.4 First return to the Iberian Peninsula

Of the 16 individuals born between 2015 and 2021 that were old enough to return to the Iberian Peninsula after their stay in the Sahel (2–3 years old), four started their return migration between late March and late April (Julian day 100 ± 17) in their second year of life, while another one returned in its third year. This bird attempted to migrate to the Iberian Peninsula in the second year but, after reaching the coast of Morocco, crossed the desert back southward without traversing the Strait of Gibraltar. The return journey took the five individuals an average of 21 ± 14 days, after which they crossed the Strait of Gibraltar and reached the Iberian Peninsula between the end of April and the beginning of June (Julian day: 121 ± 19). These individuals (one female and four males) were born in Arribes (*n* = 2), Galicia (*n* = 2), and Segovia (*n* = 1). It should be noted that the surviving individuals tagged in Arribes in 2023 (*n* = 8) are not yet old enough to begin their return to the Iberian Peninsula.

The fate of living individuals after their first return from Africa is shown in Table S1. These individuals continued to migrate regularly each autumn and remained in the Sahel until spring, when they returned to the Iberian Peninsula.

### 3.5 Mortality

**FIGURE 4:**
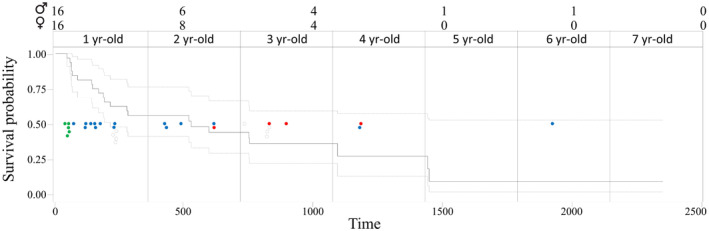
Temporal distribution of Egyptian vulture *Neophron percnopterus* that stopped transmitting GPS signals. Time zero represents the day of tagging of each bird. Above, the number of individuals (males and females) whose signal is normally received at each time is shown. The empty circles represent the time and stage (green: migration, blue: stay in the Sahel, red: stay in the Iberian Peninsula) at which each individual was lost, while the black circles indicate the age of the Egyptian vultures still emitting.

Of the 32 individuals tagged, nine were alive and transmitting at the end of this study, five of them born in 2021 (3 migrants and 2 non‐migrant birds) and four born in 2023. Of the 2‐year‐old individuals, five were lost during the first migration to the Sahel, 13 during their stay in this area and another individual was lost after returning to the Iberian Peninsula. Four individuals became untraceable at more than 2 years of age, one on its first return, delayed by 1 year, and another on its second migratory journey while in Spain. The remaining two birds (one in its fourth year and one in its sixth year) were lost in the Sahel (Figure 4 and Table 1). Of the Egyptian vultures that did not migrate, two are still alive (tagged in 2021) and another, a female, lost transmitter functionality in her fourth year but was sighted alive several months later (Figure 3; see details in Table S1). Kaplan–Meier analyses show that the rate of loss of individuals, which we assimilated to survival, was similar between individuals born in the different breeding nuclei (*χ* ^2^ = 0.62, df = 2, *p* = .73) and between males and females (*χ* ^2^ = 0.05, df = 1, *p* = .83). However, although the sample size is small, non‐migratory individuals have a slightly higher, not statistically significant, apparent survival than migratory individuals (*χ* ^2^ = 3.02, df = 1, *p* = .082).

**TABLE 1:** Number of male (m) and female (f) Egyptian vultures tagged from 2015 to 2023 as nestlings in different breeding nuclei in the Iberian Peninsula (Arribes del Duero (Arribes), Galicia and Segovia) that died during the dependence period, migration and stay in the Sahel and in the Iberian Peninsula after their first attempt to return to Europe (except one individual that attempted to return after 3 years, the others did so after 2 years). The duration of each stage (in days) and the mortality rate (deaths/duration) are shown.

Stage	Arribes		Galicia		Segovia		Total	Duration	Mortality rate
	M	F	M	F	M	F			
Dependence	0	0	0	0	0	0	0	25	0.00
Migration	2	2	0	1	0	0	5	28	0.17
Stay in the Iberian Peninsula	0	0	0	0	0	0	0	631	0.00
Stay in the Sahel	0	3	5	2	2	2	14	631	0.02
Total	7/17 (41%)^a^		8/10 (80%)		4/5 (80%)			–	–

^a^ Note that four of eight individuals tagged in 2023 (all from Arribes) are still alive as of May 2024 in the Sahel without having completed their first migratory cycle.

## 4 DISCUSSION

Our results show that juvenile Egyptian vultures born in different Spanish breeding nuclei do not differ in the duration of their dependence period, but exhibit differences in their migratory patterns. Upon arrival at the Sahel, juvenile migrants from different natal areas share a large common region until they returned to the Iberian Peninsula, usually in their second year of life. Although this was the general pattern for most of the individuals studied, several individuals of both sexes did not migrate to Africa at any time in their lives, remaining in the Iberian Peninsula in an area recently described as being used by adults and subadults of this species (Morant et al., 2020). The decision to adopt this non‐migratory strategy by juveniles may be influenced by the presence of adult conspecifics that also exhibit non‐migratory behavior. Moreover, the high densities of non‐breeding griffon vultures in this area (Delgado‐González et al., 2022) may also exert a strong heterospecific attraction (Sebastián‐González et al., 2010). This area is characterized by the abundant and predictable availability of carcasses from both wild ungulates and domestic livestock. The availability of these carcasses is permitted by EU legislation and regulation policies (CE 142/2011; Royal Decree 1632/2011) and is also linked to the relaxation of carcass disposal requirements for feeding vultures at authorized feeding points under EC 830/2005 (Arrondo et al., 2018).

Juveniles from different Spanish breeding nuclei did not show significant differences in the timing of leaving their natal territories, and these dates were similar to those recorded in other areas in northern Spain (Donázar & Ceballos, 1990). This pattern is unexpected according to latitude, as reproduction tends to be delayed the further north the population is located (Scherler et al., 2023). Local environmental conditions and population characteristics beyond latitude‐related climatology can have mutually counteracting influences on the length and timing of the dependence period (Ceballos & Donázar, 1990; Donázar & Ceballos, 1990; Martinez & Blanco, 2002; Reznikov et al., 2024). For example, Segovia has a higher abundance of predictable feeding sources (i.e., vulture restaurants; Blanco, 2014) than Galicia, which could facilitate parental food provisioning, leading to prolonged nest stays and reduced movements during this period (Reznikov et al., 2024) and affecting nestling growth and development (Donázar & Ceballos, 1989). Differences in the length of stay of juveniles in the nest could translate into variations in the length of the dependence period (Donázar & Ceballos, 1990). Yet, individual movements were similar, even between males and females, suggesting similar parental care despite potential local differences in the abundance and distribution of food. This apparent synchrony in the timing of juvenile departure from the natal territories suggests temporal constraints to migrate, which may be promoted or be a consequence of the potential advantages of association of individuals to migrate, especially for first‐time migrants (Donázar & Ceballos, 1990; Donázar et al., 1996; Phipps et al., 2019). However, although the sample size is limited, our results do not suggest that siblings show coordinated behaviors (Segovian siblings) or even similar migratory strategies (Arribes del Duero siblings), pointing to the importance of individual decisions even within family nuclei (Donázar & Ceballos, 1990).

Juveniles born in the different Spanish breeding nuclei differed in the distances travelled across the Iberian Peninsula during migration before crossing the Strait of Gibraltar. These differences were due to the varying migratory movements of individuals, with some taking direct routes and others following more circuitous paths (see Figure 1). In particular, individuals from Galicia undertook longer and more wandering journeys that covered the north‐western dehesas generally used by individuals from the other nuclei, but also large areas of the north‐eastern Iberian Peninsula. With only two breeding territories in the region, juveniles may have difficulty finding conspecifics to associate with in communal roosts and follow for direct southward migration (Donázar et al., 1996; Oppel et al., 2015). This suggests that juveniles may benefit from joining groups led by experienced individuals which can guide them along optimal routes and stopovers to minimize risks (Donázar et al., 1996; Oppel et al., 2015).

The finding that part of the juveniles did not migrate to Africa but remained in the western Iberian dehesas supports the importance of this habitat for the whole Egyptian vulture population, not only subadults and adults (Morant et al., 2020). These areas are much smaller than those used in Africa on the same dates by migratory individuals, probably because in the Iberian dehesas, there is abundant availability of sheep and cow carcasses from extensive and semi‐extensive livestock farming (Delgado‐González et al., 2022; Morant et al., 2020). In Africa, by contrast, food is more dispersed and unpredictable (Cortes‐Avizanda et al., 2011; Carrete et al., 2013), which can force individuals to travel enormous distances to meet their basic needs (Millon et al., 2019; Trierweiler et al., 2013). Surveying these vast expanses of African land poses a significant risk to the birds, as evidenced by the large number of individuals that were lost and presumed dead during this period. The strategy followed by the three juveniles from Arribes del Duero of not migrating to Africa, not only as juveniles in their first year but also in subsequent years, could increase their survival and may have an impact on the dynamics of this nucleus (Morant et al., 2022), similar to what happen in sedentary island populations (Sanz‐Aguilar et al.,^2015^ ). These non‐migratory individuals consistently used year after year the southern core (Cáceres) of the residency area in the Iberian Peninsula during the period corresponding to the stay in Africa of migratory individuals (September–March). Several communal roosts of non‐migratory individuals have been recorded in this area, with an increasing number of individuals, especially adults (about 76%) and subadults (Morant et al., 2020, 2022). Our results suggest that this non‐migratory population could originate from non‐migratory juveniles that become sub‐adults and even sedentary adults (Morant et al., 2020), although we cannot rule out that the first sedentary individuals were sub‐adults or adults who stopped migrating for some reason and attracted juveniles to do so. Morant et al. (2022) showed that some non‐migratory individuals showed migratory behavior in the following wintering season, abandoning their breeding areas to migrate to Africa, supporting the idea that this would be an individual plastic strategy. Moreover, during both prenuptial and postnuptial migration, several of the monitored Egyptian vultures stopped for variable periods of time in the same dehesas used by non‐migratory individuals, supporting the importance of this habitat for the population as a whole. Interestingly, coinciding in time with the arrival of migratory individuals to the breeding grounds and, in duration, with the breeding season, non‐migratory individuals moved consistently year after year northward, to the core area where their natal territories are located (Arribes del Duero breeding nuclei). This implies that, compared with migratory individuals remaining in the Sahel during the breeding season, non‐migratory juveniles and sub‐adults may gain some advantage for future recruitment derived from knowledge of their natal breeding areas (Morant et al., 2022; Sanz‐Aguilar et al., 2017; Serrano et al., 2021) and from obtaining public information on the quality of future breeding territories (Sergio & Penteriani, 2005). It is therefore essential to investigate in detail the factors influencing the individual decision not to migrate and to remain in the Iberian Peninsula, and whether this strategy may influence population dynamics and future trends at specific breeding nuclei. In particular, more research is needed to discern the role of phenotype, condition, health, genetics, and environment in migratory decisions and their ecological and evolutionary implications.

In general, juveniles move through shared areas in the Sahel, with no apparent segregation by sex or natal area, as has been reported for other trans‐Saharan migratory bird species (Blanco et al., 2018; García‐Macía et al., 2023; Oomen et al., 2011). These areas also overlap with those previously described for Egyptian vultures of other age classes from Western Europe (García‐Ripollés et al., 2010; Meyburg et al., 2004). This apparent panmixia may have positive implications for the exchange of knowledge and behaviors between individuals, potentially increasing the adaptive capacity and resilience of the species as a whole (Negro & Galván, 2018). However, the fact that all individuals share the same, albeit huge, area in the Sahel confronts them with common threats that affect the population as a whole (Runge et al., 2014; Webster et al., 2002).

Despite the longer and more variable movements exhibited by Galician individuals during the migration to the Strait of Gibraltar, these individuals do not seem to have a higher apparent mortality rate than those from other nuclei. It is worth mentioning that, despite the critical nature of these early stages for the survival of an individual (Newton, 2024), only one of the tracked juveniles disappeared during this period. In contrast, loss of data reception and thus apparent mortality seem to increase sharply as birds began the long journey across the Sahara Desert (Newton, 2024; Oppel, Arkumarev, et al., 2021; Oppel, Buechley, et al., 2021; Serratosa et al., 2024). Indeed, the journey across these 2500 km had the highest apparent mortality rate per unit time (5 out of the 29 individuals that started the migration to Africa were lost at this stage, which lasts an average of 11 days), suggesting that individuals rush this journey possibly because it is the most dangerous stage, mainly during their first early‐life migration, before they acquire experience (Efrat et al., 2023). In fact, all five individuals that initiated the return migration northwards completed it successfully, suggesting that the experience gained during the first migration and stay in the Sahel improved subsequent survival (Sergio et al., 2019). Previous studies have reported high mortality in this species during the pre‐adult stage using capture–recapture analysis with individually ringed nestlings (Grande et al., 2009). However, as juvenile migrants do not return from migration to Africa during the first 2–3 years of life, it was not possible to accurately estimate their survival with this methodology. Although our survival analyses are very crude and do not take into account possible transmitter failures, the data suggest that the highest mortality would occur when animals travel through the Sahara Desert and while in sub‐Saharan Africa, given the number of animals that are lost in these areas and are never contacted or seen again. After the first return to the Iberian Peninsula, and during subsequent migrations, the loss of individuals contributes to increase this high mortality rate, to the point that most of the individuals tracked in this study that could have reached the age to start prospecting future breeding areas (from 3 years of age onward) were lost earlier.

In conclusion, our results provide important insights into the migratory ecology and movement strategies of juvenile and sub‐adult Egyptian vultures from the Iberian Peninsula. A major concern is the apparent very high mortality observed during the first years of life for Egyptian vultures from Spain. Given the observed differences in migratory behavior and movements across Iberian and African regions, it is crucial to ensure the protection and availability of suitable habitats in both breeding and non‐breeding areas. Understanding the factors that influence non‐migratory behavior, observed in some individuals behavior—including genetic, phenotypic, environmental, and social factors—is essential for implementing specific conservation measures for populations that may become residents in the Iberian Peninsula over the years, as seen in some island populations (Sanz‐Aguilar et al., 2015). Based on our findings, we recommend that conservation efforts should pay particular attention to different spatial scales (given the long‐distance migration of the species and even intraspecific variation in migratory strategies) and temporal scales (taking into account that the vulnerability of the species is not the same at all stages of its development). Specifically, efforts should be directed toward protecting critical habitats along migratory routes and at wintering grounds, as well as addressing anthropogenic threats such as habitat loss and human disturbance (Oppel, Arkumarev, et al., 2021; Oppel, Buechley, et al., 2021). These measures are crucial to reduce the high pre‐adult mortality and ensure the long‐term survival of this threatened species. Research on how the sedentarization trend in the Iberian Peninsula can contribute to the conservation of the species should be a priority.

## AUTHOR CONTRIBUTIONS

**Juan Oltra:** Conceptualization (lead); data curation (lead); formal analysis (lead); investigation (equal); methodology (lead); software (lead); supervision (equal); validation (equal); visualization (lead); writing – original draft (lead); writing – review and editing (equal). **Javier García:** Conceptualization (equal); funding acquisition (equal); resources (equal); supervision (equal); validation (equal); writing – review and editing (equal). **Isidoro Carbonell:** Funding acquisition (equal); resources (equal); supervision (equal); validation (equal); writing – review and editing (equal). **José Jambas:** Funding acquisition (equal); resources (equal); supervision (equal); validation (equal); writing – review and editing (equal). **Ernesto Álvarez:** Conceptualization (equal); funding acquisition (equal); resources (equal); supervision (equal); validation (equal); writing – review and editing (equal). **Juan J. Iglesias‐Lebrija:** Conceptualization (equal); funding acquisition (equal); resources (equal); supervision (equal); validation (equal); writing – review and editing (equal). **Alberto Gil‐Carrera:** Funding acquisition (equal); resources (equal); supervision (equal); validation (equal); writing – review and editing (equal). **Juan M. Pérez‐García:** Conceptualization (lead); formal analysis (equal); funding acquisition (lead); investigation (equal); methodology (equal); project administration (lead); resources (equal); software (equal); supervision (equal); validation (equal); writing – original draft (equal). **Óscar Frías:** Conceptualization (equal); methodology (equal); resources (equal); supervision (equal); validation (equal); writing – review and editing (equal). **José L. González del Barrio:** Conceptualization (equal); methodology (equal); resources (equal); supervision (equal); validation (equal); writing – review and editing (equal). **Guillermo Blanco:** Conceptualization (lead); formal analysis (equal); funding acquisition (lead); investigation (equal); methodology (equal); project administration (lead); resources (lead); supervision (lead); validation (equal); visualization (lead); writing – original draft (lead). **Martina Carrete:** Conceptualization (lead); formal analysis (lead); funding acquisition (lead); investigation (equal); methodology (equal); project administration (lead); resources (lead); software (equal); supervision (lead); validation (equal); visualization (equal); writing – original draft (lead).

## FUNDING INFORMATION

This project was funded by of the Spanish Ministry of Science and Innovation MCIN/AEI/10.13039/501100011033 through PID2019‐109685GB‐I00 project, and IJC‐2019‐038968 contract. JO was supported by an FPI grant (PRE2020‐095280). The Ministry of Ecological Transition and Demographic Challenge of the Government of Spain as a financier through its Public Utility funds. SALORO S.L.U. funded the deployment of transmitters in the Arribes del Duero. The tagging in Segovia was possible through a research agreement contract (Ref: 060501210083) between MNCN‐CSIC and WWF Spain‐ADENA.

## CONFLICT OF INTEREST STATEMENT

We declare that we have no competing interests.

## Supporting information


Table S1

## Data Availability

All data supporting this research are available at Movebank (projects 1680287572, 316961802, and 613713666) and can be made available upon request to the authors.
