# Supplementary material for: Early life movements and mortality of Egyptian vultures: Implications for transcontinental conservation
Source: Ecol Evol. 2024 Sep 15;14(9):e70291. doi: 10.1002/ece3.70291 (PMC11402506; doi:10.1002/ece3.70291)
Supplement: Supplementary file 1 — Table S1 [file ECE3-14-e70291-s001.docx]

Table S1. Details of the Egyptian vultures *Neophron percnopterus* tagged at three breeding nuclei in the Iberian Peninsula (Arribes del Duero “Arribes”, Galicia and Segovia). For each individual, we indicate sex, transmitter model, tagging date, number of fixes downloaded (N fixes), number of monitoring days (N days), whether or not it migrated to Africa (in brackets: number of migratory events), and whether it returned or not to the Iberian Peninsula (in brackets: number of migratory events). The last column shows whether the animal was alive (normal GPS signal reception) or whether it had stopped transmitting the GPS signal and at what stage (migration to Africa, stay in Africa (Sahel) or in the Iberian Peninsula (Spain)). The latter was considered as death in the survival analysis.

| **Individual** | **Breeding nuclei** | **Sex** | **Transmitter model** | **Tagging date** | **Last tracking date** | **N fixes** | **N days** | **Migrated to Africa (N times)** | **Migrated to Iberia (N times)** | **Death stage** |
| --- | --- | --- | --- | --- | --- | --- | --- | --- | --- | --- |
| F13269 | Arribes | Male | Ornitela | 15/07/2023 | 29/08/2023 | 2151 | 45 | yes (1) | no | Migration to Africa |
| 5782 | Galicia | Female | E-obs Solar 48g GPS-GSM-GPRS | 21/07/2017 | 16/09/2017 | 3131 | 57 | yes (1) | no | Migration to Africa |
| F12264 | Arribes | Female | Ornitela | 12/07/2017 | 12/09/2017 | 1385 | 62 | yes (1) | no | Migration to Africa |
| F13265 | Arribes | Male | Ornitela | 12/07/2023 | 13/09/2023 | 3905 | 63 | yes (1) | no | Migration to Africa |
| F13270 | Arribes | Female | Ornitela | 15/07/2023 | 19/09/2023 | 1177 | 66 | yes (1) | no | Migration to Africa |
| A8A (PVC) | Segovia | Male | Ornitela | 29/07/2021 | 24/10/2021 | 98904 | 87 | yes (1) | no | Sahel |
| F4236 | Arribes | Female | Ornitela | 14/07/2021 | 04/12/2021 | 10236 | 143 | yes (1) | no | Sahel |
| F4240 | Arribes | Female | Ornitela | 12/07/2023 | 05/12/2023 | 6453 | 146 | yes (1) | no | Sahel |
| 181686 | Galicia | Male | Ornitela | 25/07/2019 | 11/01/2020 | 201 | 170 | yes (1) | no | Sahel |
| 5783 | Galicia | Female | E-obs Solar 48g GPS-GSM-GPRS | 22/07/2017 | 27/01/2018 | 29507 | 189 | yes (1) | no | Sahel |
| 370 | Galicia | Male | 45g GPS-GSM MTI | 21/07/2017 | 30/01/2018 | 48522 | 193 | yes (1) | no | Sahel |
| A8F (PVC) | Segovia | Male | Ornitela | 04/08/2021 | 08/03/2022 | 375943 | 216 | yes (1) | no | Sahel |
| F13267 | Arribes | Male | Ornitela | 14/07/2023 | 17/04/2024 | 3433 | 278 | yes (1) | no | Alive |
| A8L (PVC) | Segovia | Female | Ornitela | 09/08/2021 | 18/05/2022 | 348802 | 282 | yes (1) | no | Sahel |
| 201253 | Galicia | Female | Ornitela | 24/07/2020 | 06/05/2021 | 26848 | 286 | yes (1) | no | Sahel |
| F13268 | Arribes | Female | Ornitela | 15/07/2023 | 29/04/2024 | 4620 | 289 | yes (1) | no | Alive |
| F13266 | Arribes | Female | Ornitela | 14/07/2023 | 02/05/2024 | 5947 | 293 | yes (1) | no | Alive |
| F4239 | Arribes | Male | Ornitela | 12/07/2023 | 01/05/2024 | 6071 | 294 | yes (1) | no | Alive |
| F4234 | Arribes | Female | Ornitela | 09/07/2021 | 18/12/2022 | 35398 | 527 | yes (1) | no | Sahel |
| 9069034 | Galicia | Male | E-obs Solar 48g GPS-GSM-GPRS | 27/07/2016 | 14/01/2018 | 61463 | 536 | yes (1) | no | Sahel |
| A8C (PVC) | Segovia | Female | Ornitela | 29/07/2021 | 27/03/2023 | 532801 | 606 | yes (1) | no | Sahel |
| 6471 | Galicia | Male | E-obs Solar 48g GPS-GSM-GPRS | 06/08/2018 | 09/09/2020 | 108199 | 764 | yes (1) | no | Sahel |
| F4237 | Arribes | Male | Ornitela | 14/07/2021 | 19/08/2023 | 52800 | 766 | yes (1) | yes (1) | Spain |
| A8J (PVC) | Segovia | Male | Ornitela | 06/08/2021 | 05/02/2024 | 328990 | 913 | yes (2) | yes (2) | Alive |
| F4235 | Arribes | Female | Ornitela | 09/07/2021 | 25/04/2024 | 38594 | 1021 | yes (1) | no | Alive |
| F4238 | Arribes | Male | Ornitela | 14/07/2021 | 02/05/2024 | 58868 | 1023 | resident | - | Alive |
| F4233 | Arribes | Female | Ornitela | 04/07/2021 | 02/05/2024 | 58475 | 1033 | resident | - | Alive |
| F4232 | Arribes | Male | Ornitela | 03/07/2021 | 01/05/2024 | 36977 | 1033 | yes (2) | yes (2) | Alive |
| 9069033 | Galicia | Male | Ecotone SAKER-M | 25/07/2016 | 14/08/2019 | 230 | 1114 | yes (2) | yes (2) | Spain |
| 6470 | Galicia | Male | E-obs Solar 48g GPS-GSM-GPRS | 25/07/2019 | 05/08/2023 | 127851 | 1470 | yes (1) | no | Sahel |
| F12263 | Arribes | Female | Ornitela | 12/07/2017 | 27/07/2021 | 93283 | 1476 | resident | - | Alive |
| 9069032 | Galicia | Female | PTT-100 45g GPS/Argos Solar MTI | 30/07/2015 | 25/02/2022 | 6127 | 2402 | yes (5) | yes (4) | Sahel |

Table S2. Summary of response and exploratory variables used in the study. The table includes the variable type, range or categories, and a brief description of each variable.

| **Response variables** | **Variable Type** | **Range or Categories** | **Description** |
| --- | --- | --- | --- |
| **Date of nest departure** | Continuous | 184-245 | Julian date of leaving the nest. First time each individual moved >200 m from the nest. |
| **Date of territory departure** | Continuous | 224-254 | Julian date when individuals ceased returning to the nest. |
| **Duration of the dependence period** | Continuous | 0-51 days | Duration of the dependence period (in days), calculated from the date of nest departure to the date of territory departure. |
| **Maximum distance from the nest** | Continuous | 0.06 - 32.95 km | Maximum distance reached from the nest during the dependence period. |
| **Area covered during dependence** | Continuous | 0.1 - 787.6 km² | Total area covered during the dependence period, estimated using the 95% isopleth kernel density estimator (KDE). |
| **Time spent in Iberia** | Continuous | 2 - 59 days | Time (in days) spent in the Iberian Peninsula before migrating to sub-Saharan Africa. |
| **Migration distance in Iberia** | Continuous | 1,647 - 2,824 km | Total distance travelled during migration across the Iberian Peninsula. |
| **Time spent in Sahara Desert** | Continuous | 6 - 28 days | Time (in days) spent crossing the Sahara Desert during migration |
| **Sahel area overlap** | Continuous | 0 - 100% | Percentage overlap of areas used in the Sahel among individuals. |
| **Mortality** | Categorical | Alive, Missing, Dead | Status of the individual at the end of the study (alive, missing, dead). |
| **Exploratory variables** | **Variable Type** | **Range or Categories** | **Description** |
| **Sex** | Categorical | Male, Female | Sex of the individual, determined by molecular methods using a blood sample. |
| **Breeding nucleus** | Categorical | Arribes, Galicia, Segovia | Breeding nucleus of origin, representing different geographical areas in Spain with distinct ecological and demographic characteristics. |
| **Year of tagging** | Categorical | 2015 - 2023 | Year the individual was tagged. |
